# Improving Humanization Skills through Simulation-Based Computers Using Simulated Nursing Video Consultations

**DOI:** 10.3390/healthcare10010037

**Published:** 2021-12-26

**Authors:** Diana Jiménez-Rodríguez, Mercedes Pérez-Heredia, María del Mar Molero Jurado, María del Carmen Pérez-Fuentes, Oscar Arrogante

**Affiliations:** 1 Department of Nursing, Physiotherapy and Medicine, University of Almería, 04120 Almeria, Spain; 2 Research Management Department, Primary Care District Poniente of Almeria, Despacho: 29, 04120 Almeria, Spain; mmercedesph@yahoo.es; 3 Department of Psychology, Faculty of Psychology, University of Almería, 04120 Almeria, Spain; mmj130@ual.es (M.d.M.M.J.); mpf421@ual.es (M.d.C.P.-F.); 4 Department of Psychology, Universidad Autónoma de Chile, Providencia 7500000, Chile; 5 Red Cross University College of Nursing, Spanish Red Cross, Autonomous University of Madrid, Avenida Reina Victoria 28, 28003 Madrid, Spain; oscar.arrogante@cruzroja.es

**Keywords:** COVID-19, high fidelity simulation training, nursing education, remote consultation, telemedicine

## Abstract

During the COVID-19 confinement, we converted our clinical simulation sessions into simulated video consultations. This study aims to evaluate the effects of virtual simulation-based training on developing and cultivating humanization competencies in undergraduate nursing students. A quasi-experimental study was conducted with 60 undergraduate nursing students. A validated questionnaire was used to evaluate the acquisition of humanization competencies (self-efficacy, sociability, affection, emotional understanding, and optimism). The development of humanization competencies in this group composed of undergraduate nursing students was evaluated using virtual simulation-based training, comparing the levels obtained in these competencies at baseline (pre-test) and after the virtual simulation experience (post-test). After the virtual simulation sessions, students improved their levels in humanization total score and the emotional understanding and self-efficacy competencies, obtaining large effects sizes in all of them (rB = 0.508, rB = 0.713, and rB = 0.505 respectively). This virtual simulation modality enables training in the humanization of care with the collaboration of standardized patients in the form of simulated nursing video consultations and the performance of high-fidelity simulation sessions that comply with the requirements of best practices. Therefore, this methodology could be considered as another choice for virtual simulation. Additionally, this virtual modality could be a way to humanize virtual simulation.

## 1. Introduction

During the COVID-19 pandemic, governments around the world have declared social distancing measures to ensure the confinement of the population, including the closure of schools and universities. In this sense, this pandemic represents a challenge not only to health services but also to nursing education. In response to this exceptional situation, simulation-based education had to adapt through the use of virtual simulation modalities, thus highly increasing its use, leading to virtual simulation becoming a primary teaching strategy to provide simulated experiences [1] using online platforms, specific software or mobile devices [2,3]. Virtual simulation modalities comprise immersive simulation, screen-based simulation, serious games, virtual reality, virtual simulation/virtual patients, virtual reality simulation, and web-based simulation [4]. All these modalities provide students with near-reality, interactive virtual simulation learning experiences when face-to-face simulations are not possible [3].

To adapt our high-fidelity clinical simulation sessions to virtual simulation, we implemented simulated nursing video consultations in our university during the COVID-19 confinement [5,6]. Additionally, we considered that nursing students should practice simulated video consultations to train in this healthcare modality that has become both popular and necessary during this pandemic. In this sense, among the different telemedicine modalities, video consultations have been significantly increased [7,8], implementing them in many countries has been a digital health strategy to provide healthcare [9,10]. This modality of healthcare has multiple benefits such as avoiding agglomerations owing to social distancing restrictions, patient satisfaction, and cost reduction [11,12]. However, we were concerned about virtual interactions between nursing students and a standardized patient using virtual simulation sessions, since the distancing between them and the inability to perform an in-person consultation could lead to providing dehumanized and depersonalized nursing care training.

According to David Gaba, considered to be one of the fathers of clinical simulation, simulation is a technique not a technology [13], because simulation sessions must not be exclusively based on the use of technological equipment or devices. A simulation setting can help train students in nursing clinical skills, procedures, or techniques, and also the art of nursing generally [14]. Additionally, it can help students recognize the totality of the human being, providing patient-centered care [15]. This approach to healthcare is closely linked to the humanization of care construct [16].

Nowadays, humanization of care is a fashionable construct within healthcare services, possibly owing to society perceive they are dehumanized and depersonalized [16]. In short, humanizing healthcare means putting the human being at the center to promote and protect the health, cure diseases, or provide the best care [17]. However, there is not a consensus on the humanization of care definition to date, but most approaches to this construct offer a definition based on responding to patient’s needs [16]. The humanization of care construct implies a set of personal competencies that healthcare professionals should have to care for patients effectively and humanely [18]. In this sense, Pérez-Fuentes et al. [18] have recently proposed a humanization of care model which comprises 5 competencies required in healthcare clinical practice: optimism (to generate positive future expectations), sociability (to relate to others appropriately with assertiveness and empathy), emotional understanding (to empathize cognitively with others, placing ourselves in their place), self-efficacy (to manage successfully complex and stressful situations), and affection (to empathize emotionally with the affective state of another person).

Previous studies have demonstrated the effectiveness of simulation-based training mainly in the self-efficacy [19] and empathy [20] competencies, but no research to date has studied the effects of simulation training in all competencies required to provide humanized nursing care. Specifically, this could represent a significant challenge if this training is conducted through a virtual simulation modality, owing to the virtual interaction and distancing between nursing students and virtual patients. Therefore, this study aimed to evaluate the effects of virtual simulation-based training on developing and cultivating humanization competencies in undergraduate nursing students.

## 2. Materials and Methods

### 2.1. Research Context and Setting

A quasi-experimental study was conducted using a single-group pre-test post-test design. The development of humanization competencies in this group composed of undergraduate nursing students was evaluated using virtual simulation-based training, comparing the levels obtained in these competencies at baseline (pre-test) and after the virtual simulation experience (post-test).

### 2.2. Setting and Sample

The study was performed in a public University between 20 April and 21 May 2020, including 3rd-year undergraduate students enrolled in nursing degree (66 students). These students performed virtual simulation sessions. A total of 60 nursing students participateD in the study (90.9% response rate).

### 2.3. Simulation Design Process

All simulated nursing video consultations followed the INACSL Standards of Best Practice: Simulation^SM^ [21,22,23,24]. During these simulated sessions, all stages included in high-fidelity clinical simulation were accomplished: pre-briefing, briefing, simulated scenario, and debriefing. A virtual platform of online video conferences provided by the university (Blackboard Collaborate Launcher^TM^) was used to develop all simulation stages [5,6].

We designed six simulated scenarios related to basic healthcare at patients’ homes who presented the following clinical cases: a patient diagnosed with arterial hypertension, a post-surgical patient (laparoscopic cholecystectomy), a woman with an anxiety disorder (potential case of gender-based violence), a bed-ridden patient with a pressure ulcer, a child diagnosed with attention deficit hyperactivity disorder (ADHD), and a child with a febrile syndrome.

Besides attending to each reason for consultation, adequate management and protection measures to COVID-19 were considered, since all patients were confined during this pandemic. Standardized patients played the role of patients’ homes. These standardized patients were also facilitators during the simulated sessions, and they were changed during the different simulated scenarios. It should be noted a standardized patient played the role of caregiver in the clinical case of a bed-ridden patient with a pressure ulcer, and another played the role of mother when a child needed to be treated. To ensure a high-fidelity level of the simulation experience, we chose all standardized patients for their experience in clinical simulation methodology, and we trained them to play their roles according to recommendations by Lewis et al. [25].

All nursing students were divided into 4 groups of 12–16 students per group. In this sense, they formed 6 operational work teams of 2–3 students per group, performing a simulated scenario together and portraying the role of nursing professionals online. Meanwhile, the rest of the work teams were at home, observing their performance in their computer screen using the corresponding virtual platform for online video conferences. In this way, they could learn from the mistakes of their classmates who were performing a simulated scenario. Each simulated session lasted 4 h, and each student completed 3 simulation sessions (1 session of pre-briefing and 2 sessions where 6 simulated scenarios were performed), so each student completed a total of 12 h of simulation experience. 

### 2.4. Data Collection Instrument

To evaluate the acquisition of humanization competencies, the Healthcare Professional Humanization Scale (HUMAS) [18] was used. This questionnaire consists of 19 items with a 5-point Likert response scale (from 1 = ‘never’ to 5 = ‘always’). HUMAS comprises the 5 dimensions of humanization of care construct: self-efficacy (5 items), sociability (3 items), affection (5 items), emotional understanding (3 items), and optimism (3 items). To examine the humanization questionnaire reliability, the coefficient omega (ω) [26] was calculated. In this way, the internal consistency obtained by its creators for each dimension was satisfactory: optimism (pre-test: ω = 0.78, post-test: ω = 0.84), sociability (pre-test: ω = 0.81, post-test: ω = 0.85), emotional understanding (pre-test: ω = 0.74, post-test: ω = 0.74), self-efficacy (pre-test: ω = 0.79, post-test: ω = 0.78), affection (pre-test: ω = 0.88, post-test: ω = 0.90), and total score (pre-test: ω = 0.88, post-test: ω = 0.88). It should be noted, some items were minimally adapted since the participants were students, and not healthcare professionals (e.g., ‘I feel nervous when I am caring for my patients’ was changed by ‘I feel nervous when I think about caring for patients during my clinical practices.’ The humanization questionnaire was completed online pre- and post-virtual simulation sessions, through a link provided to the participating students.

### 2.5. Statistical Analysis

Descriptive statistics were calculated (minimal, maximal and mean scores, standard deviation, and percentages) to analyze the results obtained for demographic data and each item, subscale, and the total score obtained in HUMAS. Additionally, the coefficients omega (ω) were calculated to analyze the reliability of this questionnaire. Subsequently, the assumption of normality was tested using the Kolmogorov–Smirnov test, confirming that data did not follow a normal probability distribution. Consequently, to analyze the differences at baseline (pre-test) and after the virtual simulation experience (post-test), the Wilcoxon test was used. Additionally, to determine the effect size of the statistically significant differences obtained, the rank-biserial correlation (rB) was calculated, considering the following cut-off points: 0.10 (small), 0.30 (medium), and 0.50 (large) [27]. These data were analyzed using IBM SPSS Statistics version 24.0 software for Windows (IBM Corp., Armonk, NY, USA).

### 2.6. Ethical Considerations

This study was carried out following ethical principles for medical research of the international Declaration of Helsinki [28]. Additionally, this study was approved by the Research and Ethics Board of the Department of Nursing, Physiotherapy, and Medicine of A. University (Approval no. EFM-75/2020). All nursing students were informed about the study and who accepted to participate voluntarily, signed a written consent.

## 3. Results

A total of 60 nursing students participated in the study. The age of students ranged from 20 to 50 years (mean = 23.83; SD = 6.63). Most students were women (n = 52; 86.7%).

Descriptive data and reliabilities for each item, subscale, and the total score obtained in HUMAS at baseline (pre-test) and after virtual simulation sessions (post-test) are shown in Table 1. It should be noted that the reliability coefficients calculated for each subscale and the total score in HUMAS were quite similar to values obtained by its creators, indicating satisfactory reliability.

The mean scores obtained in each humanization dimension at baseline (pre-test) and after virtual simulation sessions (post-test) were compared (Table 2). Statistically significant differences were obtained in emotional understanding and self-efficacy dimensions, as well as in total score for the humanization scale applied, obtaining large effects sizes in all of them (rB = 0.505, rB = 0.713, and rB = 0.508 respectively).

**Figure 1:**
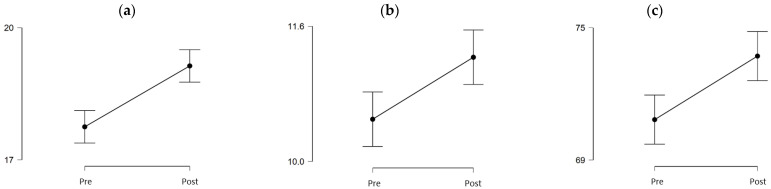
Statistically significant differences in self-efficacy and emotional understanding dimensions, and the total score obtained in HUMAS at baseline (pre-test) and after virtual simulation sessions (post-test). (**a**) Self-efficaccy, (**b**) Emotional understanding, (**c**) Total score.

Figure 1 shows graphically the magnitude of the statistically significant differences in emotional understanding and self-efficacy dimensions, and the total score obtained in HUMAS at baseline (pre-test) and after virtual simulation sessions (post-test). It should be noted that the rest of the humanization dimensions are not shown in this figure since only non-statistically significant differences were obtained.

## 4. Discussion

We converted our face-to-face simulated scenarios into a virtual format using simulated nursing video consultations in response to the closure of universities during the confinement due to the COVID-19 pandemic. We performed high-fidelity simulation sessions that complied with the requirements proposed by the INACSL Standards of Best Practice. In previous studies, nursing students expressed high satisfaction with this virtual simulation modality [5,6], perceiving that it was positively improving their learning process. However, we considered studying whether our conversion could lead nursing students to provide dehumanized and depersonalized nursing care, since virtual interactions are not the same as simulation sessions in a laboratory room.

Our results indicate the positive effects of virtual simulation-based training on developing and cultivating humanization competencies in undergraduate nursing students. After virtual simulation sessions, they improved their levels in humanization total score and the emotional understanding and self-efficacy competencies. It should be noted that emotional understanding is closely related to empathy [18]. Although there is a lack of studies analyzing the effects on the humanization of care of using clinical simulation methodology, improvements to empathy and self-efficacy in nursing students have been widely demonstrated [19,20,29]. 

Firstly, empathy is considered as the heart of all nurse-patient interactions [30], being a basic component of therapeutic relationships and a crucial factor in quality care [31]. Additionally, the positive impact of empathic healthcare interactions on patient outcomes has been widely demonstrated [31,32]. Numerous studies have demonstrated improvement to empathy levels using clinical simulation methodology [20]. Particularly, single-group studies have demonstrated a significant change in empathy between pre-test and post-test using standardized patients. However, the obtained effect sizes have been often low [30,33]. Notably, Strekalova et al. [34] used a virtual patient during simulated health history interviews and obtained emphatic responses from nursing students. In our study, we obtained increases in empathy levels and a large effect size in this humanization competency using standardized patients during virtual simulation sessions.

Regarding self-efficacy, this competency consists of a future-oriented optimistic belief that increases motivation, equating to improved performance [35]. Self-efficacy is considered as a healthcare professional’s skill in successfully managing complex and stressful situations [36]. In this sense, there is ample evidence in the literature to suggest simulation is effective at increasing this competency [19]. Specifically, single-group pre-test and post-test design studies have reported increases in self-efficacy after simulation sessions using standardized patients [37,38,39]. However, the effect sizes of simulation in self-efficacy reported by these studies are inconsistent and range from low to large. In contrast, we reported a large effect size in this humanization competence using not only standardized patients but also virtual simulation sessions.

Logically, simulated nursing video consultations mainly promote the development of non-technical skills (mainly communication skills, active listening, presence, empathy, and teamwork) [5,6]. In this sense, humanization of care and its related competencies could be included in these skills required to provide quality nursing care and decrease burnout [40]. However, while face-to-face simulation sessions usually improve technical skill performance [19,37,39], more studies are needed to analyze non-technical skill performance using virtual simulation modalities [41]. 

Lastly, although simulated nursing video consultations are not included among virtual simulation modalities in the evidence [3,4], this methodology could be considered as another choice for virtual simulation, according to their high level of fidelity in compliance with the requirements proposed by the INACSL Standards of Best Practice and the high satisfaction and positive perception expressed by nursing students in previous studies [5,6]. However, Cant et al. [3] consider clarification of the nomenclature of virtual simulation to be needed in terms of fidelity, since interactions between learners and virtual patients are different from face-to-face simulation experiences. Additionally, its use could be extended to other contexts, not only in the confinement due to the COVID-19 pandemic.

The main limitation of our study is related to the specific disadvantage of both simulated and real-life nursing video consultations: technical issues. Ensuring adequate network access and the correct functioning of virtual platforms could mitigate these potential problems [9,12]. Regarding methodological limitations, although our sample size was small, the response rate was high. Additionally, while our study did not analyze either self-efficacy or empathy using the specific validated scales, a validated scale that comprised both humanization competencies was utilized [18]. In this sense, the use of validated scales for evaluating these competencies is not consistent in the majority of the studies [19,20]. Finally, the positive effects of virtual simulation-based training on developing and cultivating humanization competencies should be confirmed by future research, so more studies are needed. These future studies should extend the sample recruited and compare it with a control group, using quasi-experimental or experimental designs and evaluating the outcomes obtained in follow-up periods (for instance, 3, 6 and/or 12 months later). Additionally, future research should also assess the acquisition of humanization of care competencies by nursing students or registered nurses using this virtual simulation modality and extend it to other settings and education centers.

## 5. Conclusions

This methodology allows nurses to be trained in the humanization of care using a virtual simulation format, in the form of simulated nursing video consultations by performing high-fidelity simulation sessions that comply with the requirements proposed by the INACSL Standards of Best Practice. Therefore, this methodology could be considered as another choice for virtual simulation. Additionally, this virtual modality allows the collaboration of standardized patients and, consequently, could be a way to humanize virtual simulation. Our results could be confirmed by future research projects using quasi-experimental or experimental designs and follow-up periods, recruiting more nursing students, including registered nurses, and extending this virtual simulation modality to other settings and education centers.

## Figures and Tables

**Table 1:** Descriptive data (minimal, maximal and mean scores, and standard deviation) and reliabilities for each item, subscale and the total score obtained in HUMAS at baseline (pre-test) and after virtual simulation sessions (post-test) (N = 60).

Items and Subscales of HUMAS	Pre-Test					Post-Test				
	Min ^1^	Max ^2^	M ^3^	SD ^4^	ω	Min ^1^	Max ^2^	M ^3^	SD ^4^	ω
Subscale 1—Optimism	4.00	15.00	11.28	2.17	0.81	6.00	15.00	11.66	2.32	0.87
1. I await the future enthusiastically.	2.00	5.00	4.13	0.87		2.00	5.00	4.23	0.90	
2. In general, I am satisfied with myself.	1.00	5.00	3.61	0.90		2.00	5.00	3.75	0.85	
3. When faced with problems, I trust that everything will come out all right in the end.	1.00	5.00	3.53	0.79		2.00	5.00	3.68	0.85	
Subscale 2—Sociability	10.00	15.00	14.40	1.15	0.79	11.00	15.00	14.50	1.03	0.87
4. In the future, when I care for patients, I will try to put myself in their place.	3.00	5.00	4.70	0.53		3.00	5.00	4.78	0.45	
5. When I start my professional career, I will give the patients or their families close, personal attention, if they need it.	3.00	5.00	4.85	0.40		4.00	5.00	4.86	0.34	
6. I will try to calm down patients and families, as I consider it an important part of caregiving.	3.00	5.00	4.85	0.44		4.00	5.00	4.85	0.36	
Subscale 3—Emotional understanding	6.00	15.00	10.50	2.07	0.77	6.00	15.00	11.23	2.09	0.70
7. When someone disrespects me, I try to understand their reasons and continue to treat that person respectfully.	2.00	5.00	3.71	0.78		1.00	5.00	3.93	0.86	
8. When I don’t like someone, I try to understand them and give them a chance for me to get to know them.	2.00	5.00	3.55	0.89		2.00	5.00	3.70	0.83	
9. When someone goes against me, I tend to analyze the situation to try and justify their behavior rationally.	1.00	5.00	3.23	0.89		1.00	5.00	3.60	0.96	
Subscale 4—Self-efficacy	5.00	24.00	17.75	2.97	0.81	11.00	25.00	19.13	2.57	0.79
10. I am able to differentiate the changes in mood in others and try to act consequently.	1.00	5.00	3.48	0.72		1.00	5.00	3.78	0.76	
11. I am satisfied with what I do and how I do it in my clinical practices.	1.00	5.00	3.80	0.75		2.00	5.00	4.01	0.59	
12. I am able to differentiate my own moods and act consequently.	1.00	5.00	3.71	0.86		1.00	5.00	3.88	0.73	
13. I think I will be prepared to cope successfully with any situation in my clinical practices.	1.00	5.00	3.15	0.79		2.00	5.00	3.53	0.72	
14. I feel that I will have a great capacity for perceiving when a patient is nor receiving adequate care.	1.00	5.00	3.60	0.80		1.00	5.00	3.91	0.67	
Subscale 5—Affection	5.00	25.00	13.10	3.48	0.86	5.00	25.00	12.81	4.18	0.89
15. When I am performing my clinical practices or I plan perform in my future career, I usually feel anxiety.	1.00	5.00	3.33	0.79		1.00	5.00	3.31	0.93	
16. I feel nervous when I think about caring for patients during my clinical practices.	1.00	5.00	2.85	0.98		1.00	5.00	3.21	0.97	
17. When in my clinical practices I perform or I think about performing clinical activities related to my future career, sometimes I feel afraid.	1.00	5.00	3.06	0.80		1.00	5.00	3.18	0.91	
18. When in my clinical practices I perform or I think about performing clinical activities related to my future career, there are situations in which I feel guilty.	1.00	5.00	3.98	0.81		1.00	5.00	3.83	1.07	
19. I feel affected when I am performing my clinical practices or I think about caring patients,	1.00	5.00	3.66	0.89		1.00	5.00	3.63	1.08	
Total score	36.00	89.00	70.83	8.66	0.89	53.00	95.00	73.71	8.07	0.86

^1^ Min.: minimal score; ^2^ Max.: maximal score; ^3^ M: mean score; ^4^ SD: standard deviation.

**Table 2:** Differences in mean scores for each humanization dimension and the total score obtained in HUMAS at baseline (pre-test) and after virtual simulation sessions (post-test) (N = 60).

Humanization Dimensions	z	*p*
Optimism	−1.68	0.091
Sociability	−0.61	0.540
Emotional understanding	−3.16 ^1^	0.002
Self-efficacy	−4.39 ^2^	0.000
Affection	−0.98	0.324
Total score	−3.28 ^1^	0.001

^1^ *p* < 0.01; ^2^ *p* < 0.001.

## Data Availability

The data presented in this study are available on request from the corresponding author.
